# 1810011o10 Rik Inhibits the Antitumor Effect of Intratumoral CD8^+^ T Cells through Suppression of Notch2 Pathway in a Murine Hepatocellular Carcinoma Model

**DOI:** 10.3389/fimmu.2017.00320

**Published:** 2017-03-22

**Authors:** Kai Dai, Ling Huang, Ya-bing Huang, Zu-bing Chen, Li-hua Yang, Ying-an Jiang

**Affiliations:** ^1^Department of Infectious Diseases, Renmin Hospital of Wuhan University, Wuhan, China; ^2^Department of Cardiology, The Central Hospital of Wuhan, Wuhan, China; ^3^Department of Pathology, Renmin Hospital of Wuhan University, Wuhan, China; ^4^Department of Hepatobiliary Surgery, Renmin Hospital of Wuhan University, Wuhan, China

**Keywords:** 1810011o10 Rik, hepatocellular carcinoma, CD8^+^ T cells, Notch2, immunity

## Abstract

The mechanisms by which tumor-responsive CD8^+^ T cells are regulated are important for understanding the tumor immunity and for developing new therapeutic strategies. In current study, we identified the expression of 1810011o10 Rik, which is the homolog of human thyroid cancer 1, in intratumoral activated CD8^+^ T cells in a murine hepatocellular carcinoma (HCC) implantation model. To investigate the role of 1810011o10 Rik in the regulation of antitumor activity of CD8^+^ T cells, normal CD8^+^ T cells were transduced with 1810011o10 Rik-expressing lentiviruses. Although 1810011o10 Rik overexpression did not influence agonistic antibody-induced CD8^+^ T cell activation *in vitro*, it inhibited the cytotoxic efficacy of CD8^+^ T cells on HCC cells *in vivo*. 1810011o10 Rik overexpression impeded CD8^+^ T cell-mediated HCC cell apoptosis and favored tumor cell growth *in vivo*. Further investigation revealed that 1810011o10 Rik blocked the nuclear translocation of Notch2 intracellular domain, which is crucial for CD8^+^ T cell activity. Furthermore, a brief *in vitro* experiment suggested that both antigen-presenting cells and TGF-β might be necessary for the upregulation of Rik expression in activated CD8^+^ T cells. In general, our study disclosed a novel mechanism underlying the negative regulation of antitumor CD8^+^ T cells during HCC progression.

## Introduction

Adaptive immune response is critical for surveillance, confinement, and elimination of malignant liver cells that give rise to hepatocellular carcinoma (HCC). Among the adaptive immune cells, both CD4^+^ T helper cells and CD8^+^ cytotoxic T cells are mostly considered to be significant players in inhibiting, impeding, and killing tumor cells (1). Furthermore, it is generally accepted that CD8^+^ T cells are the fundamental adaptive immune cells that monitor and kill tumor cells (2). However, the mechanisms by which the functions of tumor-responsive CD8^+^ T cells are regulated in tumor sites are still not thoroughly understood. Accumulative evidence suggests suppression or even loss of antitumor ability of CD8^+^ T cells in individuals with HCC (3). The suppressive factors in the tumor microenvironment might include cellular components, such as CD4^+^ regulatory T cells (4, 5), CD8^+^ regulatory T cells (6), Th17 cells (7), Kupffer cells (8), myeloid-derived suppressor cells (9, 10), and humoral components, such as IL-10 (11), TGF-β (12), and amphiregulin (13). It is of great importance to elucidate and manipulate the regulatory mechanisms of CD8^+^ T cell activity, so as to develop novel therapeutic strategies to treat HCC.

Thyroid cancer 1 (TC1), as known as C8orf4, is a 106-residue naturally disordered protein, which was originally cloned from subtractive hybridization between a papillary thyroid cancer and its surrounding normal thyroid tissue. Considered as a multi-functional protein involved in cell cycle control and transcriptional and translational regulation, TC1 has been shown to be correlated with growth and differentiation of various types of malignant cells, including thyroid cancer, ovarian carcinomas, gastric cancers, squamous cell carcinomas, and HCC (14–19). In particular, TC1 negatively regulates the self-renewal of liver cancer stem cells (19). Moreover, recent findings have suggested the regulatory role of TC1 in normal cell types such as follicular dendritic cells and hematopoietic stem/progenitor cells (20, 21). However, the role of TC1 in T cell development or function has never been reported.

In current study using a mouse HCC implantation model, we identified the expression of 1810011o10 Rik (hereinafter Rik), which is the homolog of human TC1, in intratumoral activated CD8^+^ T cells. To investigate the role of Rik in the regulation of antitumor activity of CD8^+^ T cells, normal CD8^+^ T cells were transduced with Rik-expressing lentiviruses. Although Rik overexpression did not influence agonistic antibody-induced CD8^+^ T cell activation *in vitro*, it inhibited the cytotoxic efficacy of CD8^+^ T cells on tumor cells *in vivo*. Rik-overexpressing CD8^+^ T cells were inefficient in inducing tumor cell apoptosis and suppressing tumor growth *in vivo*. In-depth investigation revealed that Rik blocked the nuclear translocation of Notch2 intracellular domain (NICD2) which is crucial for CD8^+^ T cell activity. Furthermore, a brief experiment indicated that both antigen-presenting cells and TGF-β might be necessary for the upregulation of Rik expression in activated CD8^+^ T cells. In general, our study disclosed a novel mechanism underlying the negative regulation of antitumor CD8^+^ T cells in HCC.

## Materials and Methods

### Tumor Cell Culture and Tumor Implantation Model

Mouse HCC cell lines Hepa-1c1c7, Hepa1-6, and c12 were purchased from American Type Culture Collection and were maintained in Dulbecco’s modified Eagle’s medium supplemented with 10% fetal bovine serum in a 37°C humidified incubator.

All animal experiments were conducted in compliance with institutional guidelines and Wuhan University Guidelines for the Use of Animals. All animal procedures were approved by the Wuhan University School of Medicine Animal Care and Use Committee. C57BL/6 and BALB/c mice were purchased from Vital River Laboratories (Beijing, China). Rag1^−/−^ mice (C57BL/6 background) were purchased from Shanghai Model Organisms Center, Inc. Six- to eight-week-old male mice were used in all experiments. All mice were housed under controlled temperature and light conditions following the Institutional Animal Care guidelines. For tumor implantation, 3 × 10^6^ HCC cells in 60 μl of phosphate-buffered saline (PBS) were subcutaneous injected into the dorsal skin of immunocompetent C57BL/6 mice or Rag1^−/−^ mice.

### Isolation of T Cells from Tumor Implants

Briefly, mice were euthanized by inhalation of carbon dioxide for an average of 5 min, followed by perfusion with 20 ml of ice-cold PBS through the right ventricle of the heart. The subcutaneous tumor implant was taken and minced into small pieces with surgical scissors before incubation on an orbital shaker for 30 min at 37°C in 5 ml of RPMI medium containing 1 mg/ml Collagenase IV (Thermo Fisher Scientific, 17104019), 4 mM CaCl_2_, and 10% fetal bovine serum (Hyclone, SH30088.02). After that, the tumor tissue was pressed through a 70-μm nylon mesh and centrifuged at 250 *g* for 5 min. The tissue pellet was resuspended in 5 ml of RPMI medium and overlaid onto 70% Percoll solution (GE Healthcare, 17-0891-09). After 15 min of centrifugation at 500 *g*, cells in the interlayer were collected, washed twice with PBS, and subject to further experiments. In some experiments, cells isolated from six to eight tumor implants were pooled for investigation.

### Flow Cytometry

To analyze the splenic and intratumoral T cells, the following antibodies were used: PE anti-CD3 (561799), PE anti-CD4 (561829), APC-Cy7 anti-CD8a (561967), FITC anti-CD44 (553133), Percp-Cy5 anti CD62L (560513), PE anti-TNF-α (561063), APC anti-CD45 (561018), APC anti-CD31 (561814), PE anti-Notch1 (562754), PE anti-Notch2 (562755), FITC Annexin V (556419), and propidium iodide (556463) were from BD Bioscience. APC anti-TCRβ (17-5961), PE-Cy7 anti-granzyme B (25-8898), PE anti-perforin (12-9392), and FITC anti-Ki67 (11-5698) were ordered from eBioscience. All antibodies were 1:100 diluted before use unless specified. For cell surface staining, cells were incubated in antibody-containing PBS for 15 min on ice before test on a Beckman Coulter Cytomics FC500 Flow Cytometry Analyzer. Dead cells were excluded with 1 μg/ml propidium iodide. For intracellular cytokine and Ki67 staining, cells were fixed with 4% paraformaldehyde for 15 min at room temperature, permeabilized with 90% ice-cold methanol for 30 min on ice and were then stained at room temperature for 1 h with corresponding cytokine antibodies. The data were analyzed using a Cell Quest Pro software. Cell sorting was done by a BD FACSAria cell sorter.

### RNA Extraction, cDNA Synthesis, and Quantitative Polymerase Chain Reaction

Cells or tissues were lysed in Trizol reagent (Thermo Fisher Scientific, 15596026), and RNAs were extracted following the manufacturer’s protocol. Two micrograms of RNA from each sample were used for cDNA synthesis using the SuperScript^®^ III First-Strand Synthesis System (Thermo Fisher Scientific, 18080051) following the manufacturer’s protocol. qRT-PCR was conducted using Fast SYBR^®^ Green Master Mix (Thermo Fisher Scientific, 4385610) on a StepOnePlus™ Real-time PCR System (Thermo Fisher Scientific, 4376374). Primer pairs used for amplification are as follows: β-actin (5′-AGAGGGAAATCGTGCGTGAC-3′ and 5′-CAATAGTGATGACCTGGCCGT-3′); TNF-α (5′-GCCTCTTCTCATTCCTGCTTG-3′ and 5′-CTGATGAGAGGGAGGCCATT-3′); perforin (5′-CTGGCAGGGACGATGACCT-3′ and 5′-GGGAACCAGACTTGGGAGC-3′); granzyme B (5′-ATCAAGGATCAGCAGCCTGA-3′ and 5′-TGATGTCATTGGAGAATGTCT-3′); HES1 (5′-ATAGCTCCCGGCATTCCAAG-3′ and 5′-GTATTTCCCCAACACGCTCG-3′); HES6 (5′-AAGCCCCTGGTGGAGAAGAA-3′ and 5′-TTGGCCTGCACCTCGGTA-3′); and HEY1 (5′-GTGGGAAAGGGATGGTTGAG-3′ and 5′-GAGGAGTTAACTGCAGTGGC-3′). Relative abundance of RNA was analyzed using 2^−ΔΔCt^ method.

### Immunoblot

Extraction of whole cell proteins was conducted using RIPA buffer (20 mM Tris–HCl, pH 7.5, 150 mM NaCl, 1 mM Na_2_EDTA, 1 mM EGTA, 1% NP-40, 1% sodium deoxycholate) containing protease inhibitors (Sigma-Aldrich, SRE0055-1BO). Extraction of cytosolic and nuclear proteins was conducted using the ReadyPrep protein extraction kit (Bio-Rad, 1632089) according to the manufacturer’s manual. Proteins were quantified using Pierce 660 nm Protein Assay Reagent (Thermo Fisher Scientific, 22660). Immunoblot was performed using the protocol as previously described (22). The lysates were loaded onto 4–20% Mini-PROTEAN^®^ TGX™ Precast Gels (Bio-Rad, 4561091). The following antibodies were used: anti-β-actin (Santa Cruz Biotechnology, sc-130300), anti-TC1 (Santa Cruz Biotechnology, sc-98165), anti-caspase-3 (Cell Signaling Technology, 9662), anti-cleaved caspase-3 (Cell Signaling Technology, 9664), anti-Notch1 (Cell Signaling Technology, 4380), anti-Notch2 (Santa Cruz Technology, sc-5545), anti-NICD1 (Cell Signaling Technology, 4147), and anti-NICD2 (Sigma-Aldrich, SAB4502022). The dilutions of the antibodies were based on the manufacturers’ instructions. The membranes were developed using SuperSignal West Femto Chemiluminescent Substrate (Thermo Fisher Scientific, 34095). The optical density was analyzed using a BioSpectrum 500 imaging system (Ultra-Violet Products Ltd.).

### Immunoprecipitation

CD8^+^ T cells were lysed in 50mM Tris–HCl (pH7.4), 150mM NaCl, 0.5% NP-40, and protease inhibitors (Sigma-Aldrich, SRE0055-1BO) for 30 min on ice followed by centrifugation at 3,000 *g* for 5 min at 4°C. The supernatant containing a total of 500 μg proteins was collected for immunoprecipitation. One milligram of Dynabeads™ Protein A (Thermo Fisher Scientific, 10001D) was coupled to 5 mg anti-TC1 (Santa Cruz Biotechnology, sc-98165) antibody following the manufacturer’s manual. The supernatant was mixed with 1 mg anti-TC1 antibody-coupled Dynabeads, incubated overnight at 4°C on a rotator, and eluted in a low pH buffer. The elution was mixed with 2× Laemmli buffer before being boiled for 5 min and was then loaded onto an 8% SDS-PAGE gel for Immunoblot with anti-NICD2 antibody (Sigma-Aldrich, SAB4502022).

### Immunofluorescent Staining

Cell suspensions were air dried on poly-l-lysine-coated glass slides before fixation in 4% paraformaldehyde for 15 min at room temperature. The cells were then washed three times in PBS and blocked with PBS containing 10% goat serum and 0.1% Triton X-100 for 1 h. Anti-Notch2 (Santa Cruz Technology, sc-5545) antibody was diluted in 0.1% Triton X-100-PBS and was used to incubate cells overnight at 4°C. Cells were then washed three times in PBS followed by incubation with FITC-conjugated goat anti-rabbit IgG (Abcam, ab6717) for 1 h at room temperature. Cells were then washed two times in PBS and mounted with ProLong^®^ Gold Antifade Mountant (Thermo Fisher Scientific, P36930). Cells were observed on an Olympus BX51 fluorescence microscope.

### Terminal Deoxynucleotidyl Transferase dUTP Nick End Labeling (TUNEL)

The mice were sacrificed and perfused with 30 ml of 10% formalin. Tumor implants were taken for further fixation in 10% formalin overnight, followed by routine processing of dehydration and paraffin embedding. The 5-μm sections were prepared on a HMT-2258 Manual Rotary Microtome. TUNEL was performed using the DeadEnd™ Fluorometric TUNEL System (Promega, G3250) following the manufacturer’s recommendations. The sections were observed on an Olympus BX51 fluorescence microscope.

### Enrichment of Splenic CD8^+^ T Cells and Depletion of T Cells from Splenocytes

Splenic CD8^+^ T cells were enriched from mice at day 10 to day 12 after HCC inoculation using the MojoSort™ Mouse CD8 T Cell Isolation Kit (Biolegend, 480008) following the manufacturer’s manual. Depletion of T cells from splenocytes was done through negatively selection using CD3 staining and flow cytometry sorting.

### *In Vitro* T Cell Culture and Treatment

CD8^+^ T cells were cultured at the cell density of 1 × 10^6^/ml in supplemented RPMI 1640 (containing 10% fetal bovine serum, 2 mM l-glutamine, 100 U/ml penicillin, and 100 μg/ml streptomycin) before further experiments. For coculture of T cells with tumor cells, 2.5 × 10^4^ Hepa-1c1c7 cells were cultured in each well of a 96-well round-bottom culture plate for 6 h, followed by addition of 2.5 × 10^4^ CD8^+^ T cells and incubated for another 24 h. Floating cells were collected, and adherent cells were then lifted and flushed by 0.25% Trypsin-EDTA (Thermo Fisher Scientific, 25200056) for 3 min at 37°C. Floating and flushed adherent cells were pooled for CD45 and Annexin V staining.

For lentiviral transduction, a 24-well culture plate was coated with 5 μg/ml anti-CD3 monoclonal antibody (eBioscience, 16-0032-82). A total of 1 × 10^6^ sorted CD8^+^ T cells were seeded into each well in the presence of 2 μg/ml anti-CD28 antibody (eBioscience, 16-0281-81) and 100 U/ml recombinant mouse IL-2 (eBioscience, 14-8021-64). At day 4 of stimulation, 5 μg/ml polybrene and 1810011O10 Rik lentiviral vector (MOI: 20, abm Inc., LV442947) were added into the culture. After overnight incubation, cells were washed with medium and stimulated the same way for additional 3 days. In some experiments, CD8^+^ T cells were labeled with 5 μM 5-(and 6)-carboxyfluorescein diacetate succinimidyl ester (CFSE, eBioscience, 65-0850-84) before stimulation. For intracellular staining, cells were treated with 20 ng/ml phorbol ester (Sigma-Aldrich, P1585-1MG) and 1 μM ionomycin (Sigma-Aldrich, I0634-1MG) with 5 μg/ml brefeldin A (Sigma-Aldrich, B5936-200UL) and 5 μg/ml monensin (Sigma-Aldrich, M5273-500MG) for the last 4 h of stimulation.

For Notch signaling detection, a 24-well culture plate was coated with 10 μg/ml recombinant mouse delta-like 1 (DLL1) Fc chimera protein (R&D Systems, 5026-DL) overnight at 4°C. A total of 2 × 10^6^ sorted CD8^+^ T cells were seeded into each well and incubated for 1 h before immunoblot and 4 h before RNA extraction, respectively.

For coculture of CD8^+^ T cells with T cell-free splenocytes, T cell-free splenocytes were acquired from BALB/c mice and were treated with 25 μg/ml Mitomycin C (Sigma-Aldrich, M4287-2MG) at 37°C for 30 min. Then, splenocytes were washed three times with PBS and 2 × 10^6^ splenocytes were plated into each well of a 24-well culture plate. A total of 2 × 10^6^ CD8^+^ T cells were sorted from C57BL/6 mice and were plated into each well. In some wells, recombinant mouse TGF-β (R&D systems, 7666-MB-005) was added into culture at 10 ng/ml. The plate was centrifuged at 250 *g* for 5 min. Cells were incubated for 6 days. Then, the whole culture was stained with APC anti-CD3 antibody, and CD3^+^ T cells were sorted by flow cytometry for Immunoblot.

### Adoptive Transfer

To track the recruitment of Rik-overexpressing CD8^+^ T cells, these cells were labeled with CellTrace Far Red (Thermo Fisher Scientific, C34572) according to the manufacturer’s manual. A total of 5 × 10^6^ labeled Rik-overexpressing CD8^+^ T cells and 5 × 10^6^ unlabeled control CD8^+^ T cells were mixed in 100 μl of PBS before infusion into tumor-bearing Rag1^−/−^ mice through retro-orbital injection. Three days after transfer, intratumoral T cells from individual mouse were pooled and analyzed.

To analyze the role of Rik in CD8^+^ T cell-mediated antitumor function, at day 21 after tumor inoculation in Rag1^−/−^ mice, 1 × 10^7^ CD8^+^ T cells in 100 μl of PBS were transferred into each mouse *via* retro-orbital injection. The injection was conducted twice a week for two consecutive weeks. One week after the last injection, the mice were sacrificed for evaluation of tumor growth. The tumor volume was measured according to the standard formula 1/2 × *L* × *W*^2^.

### Statistical Analysis

Quantitative data were expressed as mean ± SEM from the indicated number of experiments. Student’s *t*-test or one-way ANOVA was used for comparison of mean between the groups. *p* Values <0.05 were considered significant.

## Results

### Rik Is Predominantly Expressed in Intratumoral Activated CD8^+^ T Cells

**Figure 1:**
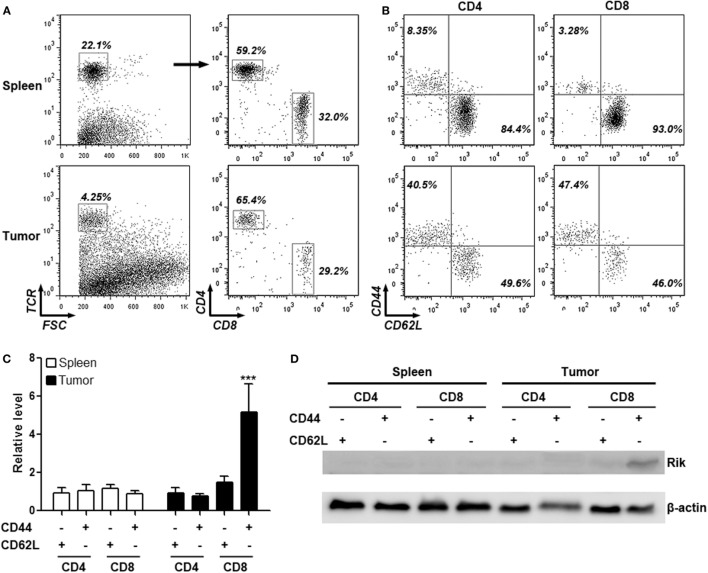
**Intratumoral CD8^+^CD44^+^CD62L^−^ T cells express Rik**. **(A)** Flow cytometry gating strategy of splenic and intratumoral CD4^+^ and CD8^+^ T cells. Total T cells were gated based on TCR staining and were subsequently discriminated by CD4 and CD8 staining. Numbers in the plots are proportions of gated cell populations. **(B)** Representative dot plots showing expression of CD44 and CD62L on splenic and intratumoral CD4^+^ and CD8^+^ T cells. Numbers in the plots are proportions of gated cell populations. **(C)** Rik mRNA level in splenic and intratumoral CD4^+^ and CD8^+^ T cells. *N* = 5 per group. **(D)** Rik protein expression in splenic and intratumoral CD4^+^ and CD8^+^ T cells. This is a representative image of two independent experiments. ***p < 0.001 compared with splenic CD4^+^CD44^−^CD62L^+^ cells.

To determine the expression of Rik in intratumoral T cells, mice were inoculated with Hepa-1c1c7 cells and intratumoral T cells were isolated for analysis. In immunocompetent C57BL/6 mice, subcutaneously injected Hepa-1c1c7 cells formed tumor implants 1 week after inoculation, but these tumor implants disappeared within 2 weeks after inoculation, probably being eradicated by antitumor immune reactions. Therefore, intratumoral T cells were analyzed between day 8 and day 10 post-inoculation when tumor implants still existed. Analysis of the activation markers demonstrated that over 40% of either CD4^+^ or CD8^+^ intratumoral T cells were phenotypically activated T cells (CD44^+^CD62L^−^), in comparison with the low frequency of splenic activated T cells (Figures 1A,B). qRT-PCR assay indicated that intratumoral CD8^+^CD44^+^CD62L^−^ T cells expressed substantially higher Rik mRNA level than all other splenic and intratumoral T cell subpopulations (Figure 1C). Consistently, Rik protein was predominantly detected in intratumoral CD8^+^CD44^+^CD62L^−^ T cells (Figure 1D). This phenomenon was also seen in tumor implants formed by other two HCC cell lines (Figure S1 in Supplementary Material). Since Hepa-1c1c7 cells formed relatively larger tumor implants than the other two cell lines (data not shown), we chose Hepa-1c1c7 cells for the following experiments.

### CD8^+^CD44^+^CD62L^−^ T Cells Are HCC-Reactive T Cells

**Figure 2:**
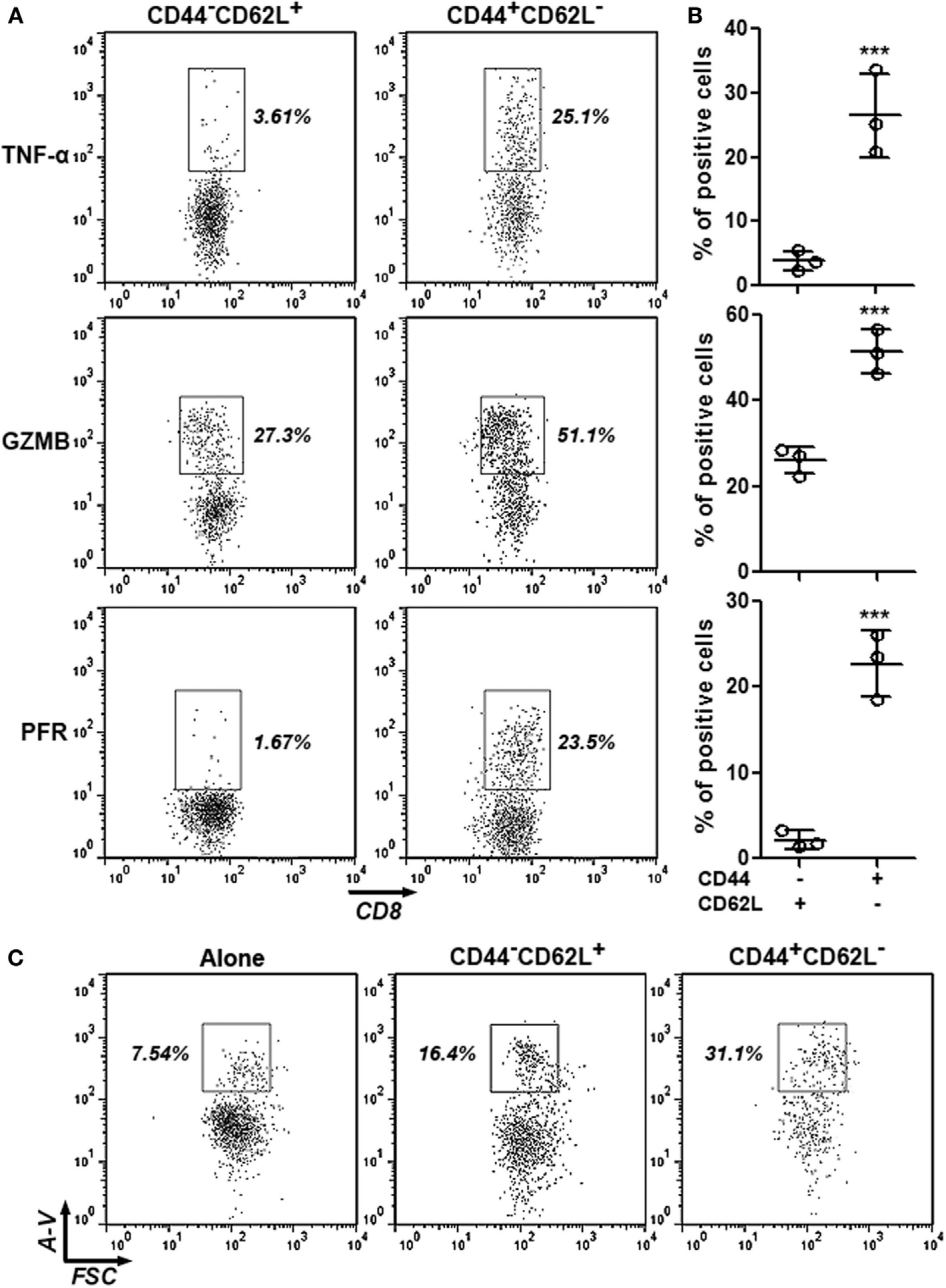
**Cytotoxic activity of intratumoral CD8^+^CD44^+^CD62L^−^ and CD8^+^CD44^−^CD62L^+^ T cells**. **(A,B)** Expression of TNF-α, granzyme B, and perforin in intratumoral CD8^+^ T cell subpopulations was analyzed by intracellular staining. Representative dot plots were shown in panel **(A)**, and statistical analysis was demonstrated in panel **(B)**. *N* = 3 per group. **(C)** Hepa-1c1c7 cell apoptosis after 24-h coculture with intratumoral CD8^+^ T cell subpopulations. The whole cells were stained with APC anti-CD45 antibody and FITC Annexin V. Only CD45-negative tumor cells were shown in the dot plots. This is a representative image of three independent experiments. Numbers in the plots are proportions of gated cell populations. Alone: tumor cells cultured alone. CD44^−^CD62L^+^: tumor cells cocultured with CD8^+^CD44^−^CD62L^+^ T cells. CD44^+^CD62L^−^: tumor cells cocultured with CD8^+^CD44^+^CD62L^−^ T cells. A-V, Annexin V (***p < 0.001).

To ascertain the anti-HCC activity of intratumoral CD8^+^CD44^+^CD62L^−^ T cells, expression of cytotoxic mediators in intratumoral CD8^+^CD44^−^CD62L^+^ and CD8^+^CD44^+^CD62L^−^ T cells were assessed through intracellular staining. Very few CD8^+^CD44^−^CD62L^+^ T cells expressed TNF-α and perforin, whereas approximately 25% of CD8^+^CD44^−^CD62L^+^ T cells expressed granzyme B. However, the frequencies of CD8^+^CD44^+^CD62L^−^ T cells which expressed these cytotoxic mediators were remarkably higher (26% expressing TNF-α, 50% expressing granzyme B, and 22% expressing perforin), suggesting that CD8^+^CD44^+^CD62L^−^ T cells were cytotoxic T cells (Figures 2A,B). To further test their reactivity to HCC, these T cell subpopulations were cocultured with Hepa-1c1c7 cells before Hepa-1c1c7 apoptosis was evaluated. As shown in Figure S2 in Supplementary Material and Figure 2C, CD8^+^CD44^−^CD62L^+^ T cells moderately induced Hepa-1c1c7 cell apoptosis in comparison to Hepa-1c1c7 cells cultured alone, while CD8^+^CD44^+^CD62L^−^ T cells robustly caused Hepa-1c1c7 cell apoptosis. These data indicated that CD8^+^CD44^+^CD62L^−^ T cells were potent HCC killers.

### Rik Does Not Alter Agonistic Antibody-Induced CD8^+^ T Cell Activation *In Vitro*

**Figure 3:**
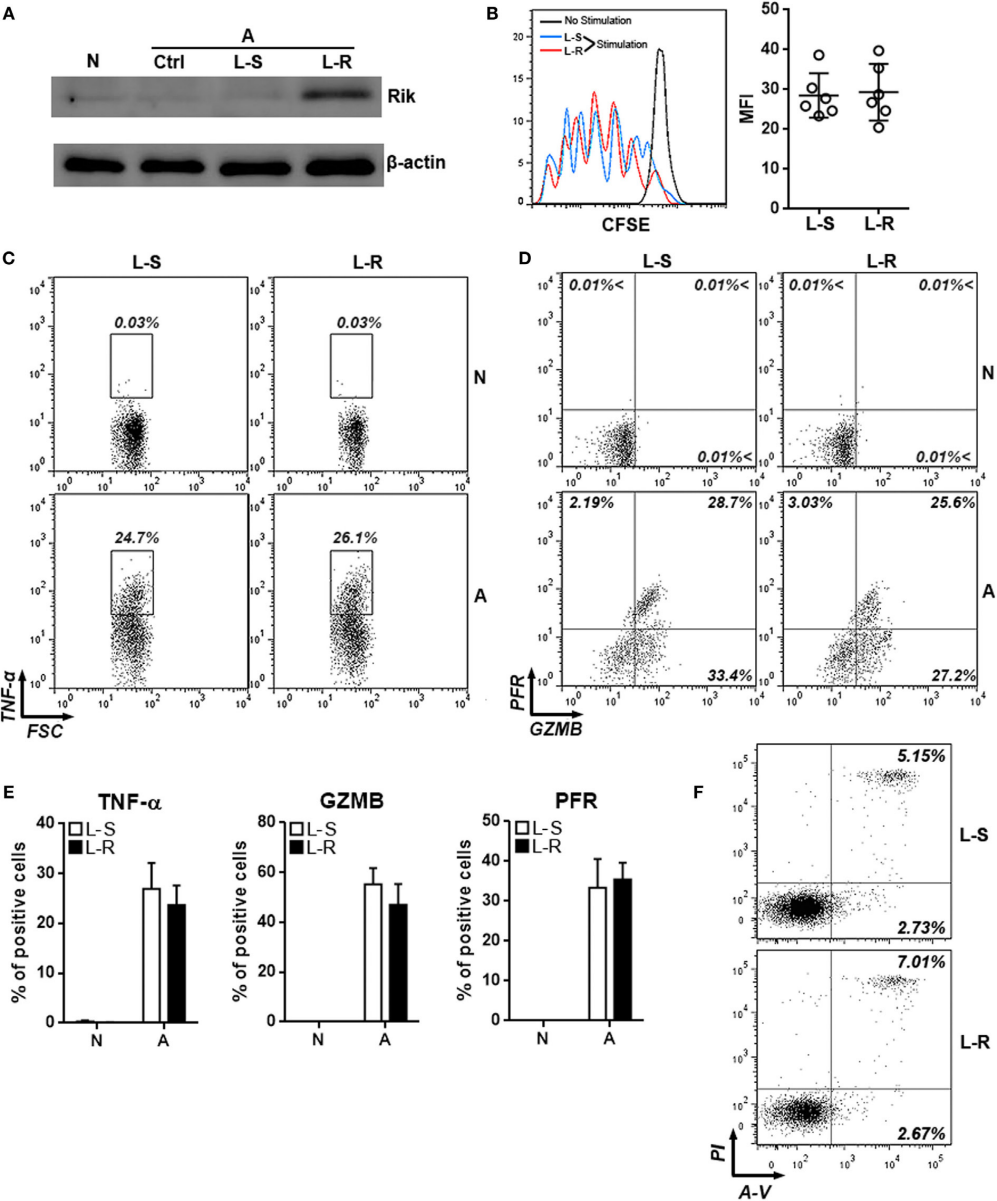
**Overexpression of Rik does not influence CD8^+^ T cell activation *in vitro***. **(A)** Expression of Rik after lentiviral transduction. N: naïve CD8^+^ T cells. A: agonistic antibody-stimulated CD8^+^ T cells. Ctrl: no transduction. L-S: transduction with lentiviruses containing scramble sequence. L-R: transduction with lentiviruses containing Rik sequence. This is a representative image of two independent experiments. **(B)** CFSE dilution in lentivirus-transduced CD8^+^ T cells. Left panel: representative histograms. Right panel: statistics of mean fluorescent intensity. **(C,D)** Intracellular staining of TNF-α **(C)**, granzyme B and perforin **(D)** in lentivirus-transduced CD8^+^ T cells. Numbers in the plots are proportions of gated cell populations. N: naïve CD8^+^ T cells. A: agonistic antibody-stimulated CD8^+^ T cells. L-S: transduction with lentiviruses containing scramble sequence. L-R: transduction with lentiviruses containing Rik sequence. **(E)** Statistical analysis of the proportions of CD8^+^ T cells expressing TNF-α, granzyme B, and perforin. *N* = 5 per group. **(F)** CD8^+^ T cell apoptosis after stimulation and lentiviral transduction. A-V, Annexin V; PI, propidium iodide. This is a representative image of two independent experiments.

To evaluate the role of Rik in CD8^+^ T cell activation, splenic CD8^+^ T cells isolated from tumor-bearing mice were activated with immobilized anti-CD3 and soluble anti-CD28 antibodies and were subsequently transduced with lentiviruses expressing Rik. Overexpression of Rik was substantiated by Immunoblot (Figure 3A; Figure S3 in Supplementary Material). Interestingly, agonistic antibody-induced CD8^+^ T cell activation itself could not increase Rik expression (Figure 3A), suggesting that CD3/CD28 signaling were not sufficient to induce Rik expression. Furthermore, Rik overexpression did not impact T cell proliferation (Figure 3B). In naive T cells, transduction of lentiviruses with or without Rik sequence did not upregulate production of TNF-α, granzyme B, and perforin (Figures 3C–E), suggesting that lentiviral transduction had no effect on expression of cytotoxic mediators in resting CD8^+^ T cells. Notably, in comparison with control lentiviruses (L-S), transduction of Rik-expressing lentiviruses (L-R) did not change the expression of TNF-α, granzyme B, and perforin in activated CD8^+^ T cells (Figures 3C–E). Moreover, Rik overexpression did not remarkably alter CD8^+^ T cell apoptosis (Figure 3F). Taken together, our data indicated that Rik expression did not influence agonistic antibody-induced CD8^+^ T cell activation.

### Rik Inhibits Antitumor Activity of CD8^+^ T Cells *In Vivo*

**Figure 4:**
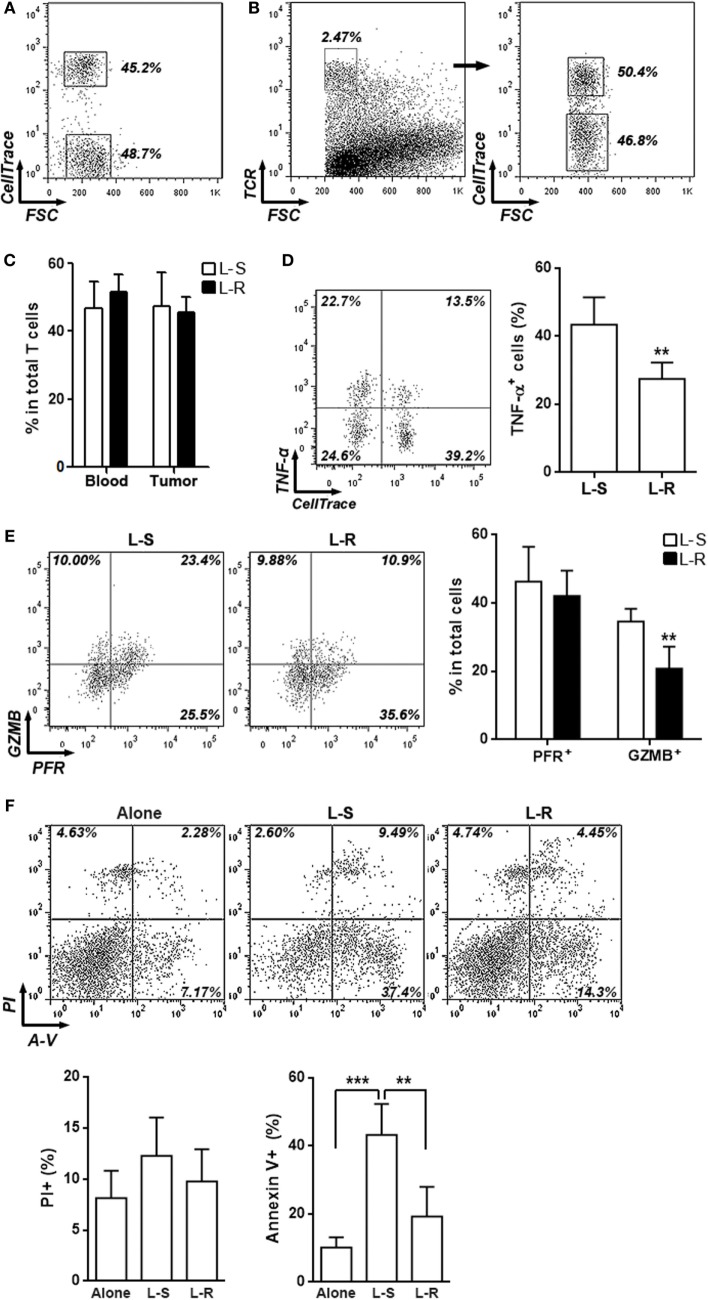
**Rik inhibits cytotoxic activity of activated CD8^+^ T cells *in vivo***. **(A)** CD8^+^ T cell mixture before adoptive transfer. CD8^+^ T cells transduced with Rik-expressing lentiviruses were labeled with CellTrace Far Red and were then mixed with equivalent number of unlabeled CD8^+^ T cells transduced with lentiviruses containing scramble sequence. Proportion of each CD8^+^ T cell subpopulation was shown in the dot plot. **(B,C)** Proportions of labeled and unlabeled CD8^+^ T cells in tumor implants. Intratumoral T cells were distinguished by TCR staining and CellTrace Far Red signal. Representative dot plots were shown in panel **(B)**, and statistical analysis was shown in panel **(C)**. L-S: CD8^+^ T cells transduced with lentiviruses containing scramble sequence. L-R: CD8^+^ T cells transduced with lentiviruses containing Rik sequence. Numbers in the plots are proportions of gated cell populations. *N* = 3 per group. **(D,E)** Expression of TNF-α **(D)**, granzyme B and perforin **(E)** in transferred CD8^+^ T cells isolated from tumor implants. Note that these cells were not stimulated *in vitro* with phorbol ester. Left panels: representative dot plots. Right panels: statistics. Numbers in the plots are proportions of gated cell populations. *N* = 6 per group. **(F)** Hepa-1c1c7 cell apoptosis after 24-h coculture with transferred CD8^+^ T cells isolated from tumor implants. Alone: tumor cells cultured alone. L-S: tumor cells cocultured with control CD8^+^ T cells. L-R: tumor cells cocultured with Rik-overexpressing CD8^+^ T cells. Upper panel: representative dot plots. Lower panel: statistics of PI^+^ or Annexin V^+^ tumor cells. *N* = 4 per group (**p < 0.01; ***p < 0.001).

To assess the effects of Rik on CD8^+^ T cell function *in vivo*, Rik-overexpressing CD8^+^ T cells were labeled with CellTrace Far Red and were 1:1 mixed with unlabeled control CD8^+^ T cells (Figure 4A). Then, the T cell mixture was adoptively transferred into recipient Rag1^−/−^ mice which had Hepa-1c1c7 tumor implants. Three days after transfer, intratumoral T cells were analyzed. The ratio between CellTrace Far Red-positive and -negative intratumoral T cells was close to 1:1 (Figures 4B,C), suggesting that Rik overexpression did not change recruitment of CD8^+^ T cells into tumor sites. Interestingly, production of TNF-α and granzyme B was decreased in Rik-overexpressing CD8^+^ T cells, while perforin expression was not changed (Figures 4D,E). Consistent with the downregulation of cytotoxic mediators, when sorted Rik-overexpressing CD8^+^ T cells were cocultured with Hepa-1c1c7 cells, they induced less Hepa-1c1c7 apoptosis than control CD8^+^ T cells did (Figure 4F). Taken together, Rik impaired cytotoxic activity in Hepa-1c1c7 implants.

### Rik Expression in CD8^+^ T Cells Is Beneficial for Tumor Survival

**Figure 5:**
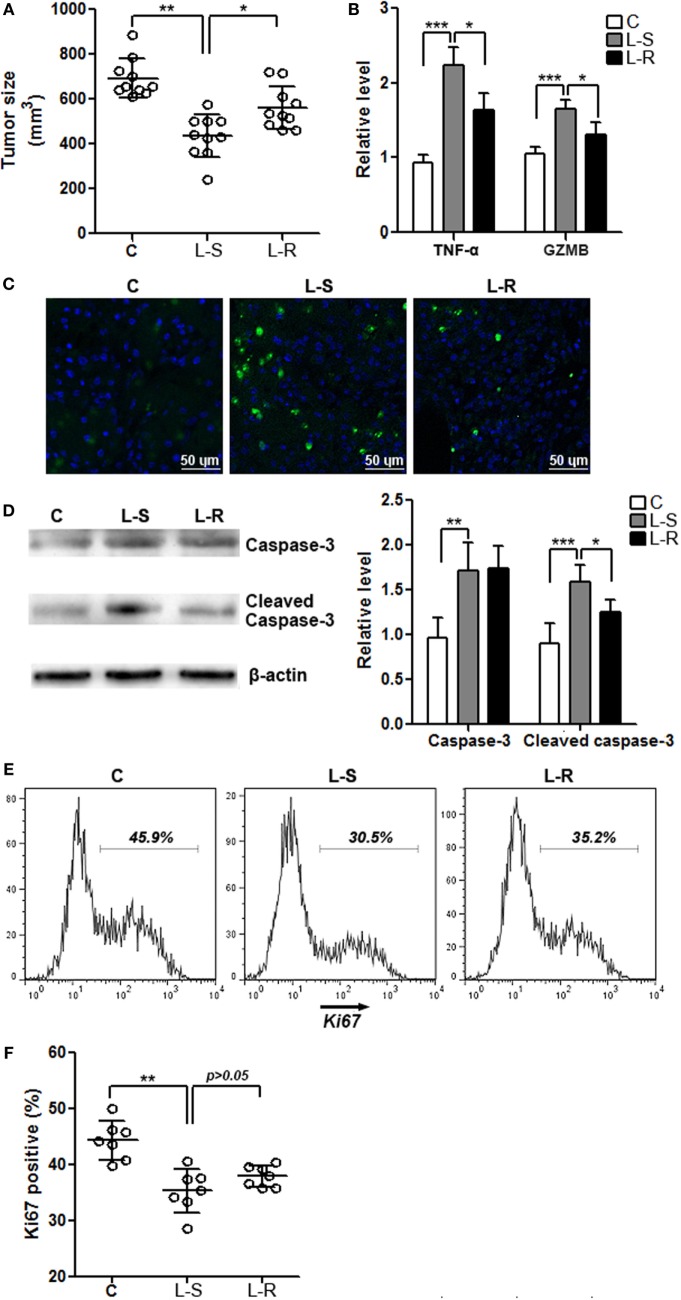
**Rik expression in CD8^+^ T cells favors tumor survival**. **(A)** Tumor size. C: control mice receiving phosphate-buffered saline. L-S: mice receiving CD8^+^ T cells transduced with scramble lentiviruses. L-R: mice receiving CD8^+^ T cells transduced with Rik-expressing lentiviruses. Each circle represents an individual mouse. **(B)** mRNA levels of TNF-α and granzyme B in tumor tissues. *N* = 4 per group. **(C)** Tumor cell apoptosis is indicated by terminal deoxynucleotidyl transferase dUTP nick end labeling. This is a representative of three independent experiments. **(D)** Activation of caspase-3 in tumor tissues. Left panel: representative Immunoblot image. Right panel: statistics of caspase-3. *N* = 5 per group. **(E,F)** Tumor cell proliferation is demonstrated by Ki67 staining. Tumor implants were digested as described in Section “Materials and Methods.” Then the whole tissue was pressed through a 70-μm nylon mesh to prepare a single cell suspension, followed by staining with APC anti-CD45 and APC anti-CD31 antibodies. Cells were then stained for Ki67 as described in Section “Materials and Methods.” CD45^−^CD31^−^ tumor cells were shown here. Representative histograms are shown in panel **(E)**, and statistical analysis for Ki67^+^ cells were shown in panel **(F)**. *N* = 7 per group (*p < 0.05; **p < 0.01; ***p < 0.001).

Control CD8^+^ T cells and Rik-overexpressing CD8^+^ T cells were periodically transferred into recipient Rag1^−/−^ mice that were bearing Hepa-1c1c7 tumor implants, respectively. One week after the last cell transfer, evaluation of tumor size indicated larger tumors in mice receiving Rik-overexpressing CD8^+^ T cells, compared with tumors in mice receiving control CD8^+^ T cells (Figure 5A). In mice receiving Rik-overexpressing CD8^+^ T cells, TNF-α and granzyme B levels in tumor implants were less than those in mice receiving control CD8^+^ T cells (Figure 5B). Consistently, TUNEL demonstrated less apoptotic tumor cells in mice receiving Rik-overexpressing CD8^+^ T cells (Figure 5C), and less caspase-3 activation was also observed in the same group (Figure 5D). With regard to tumor cell proliferation, although there was a trend of increase of Ki67-positive tumor cells in mice receiving Rik-overexpressing CD8^+^ T cells relative to mice receiving control CD8^+^ T cells, the change was statistically insignificant (Figures 5E,F).

### Rik Inhibits Nuclear Translocation of Cleaved Notch2

**Figure 6:**
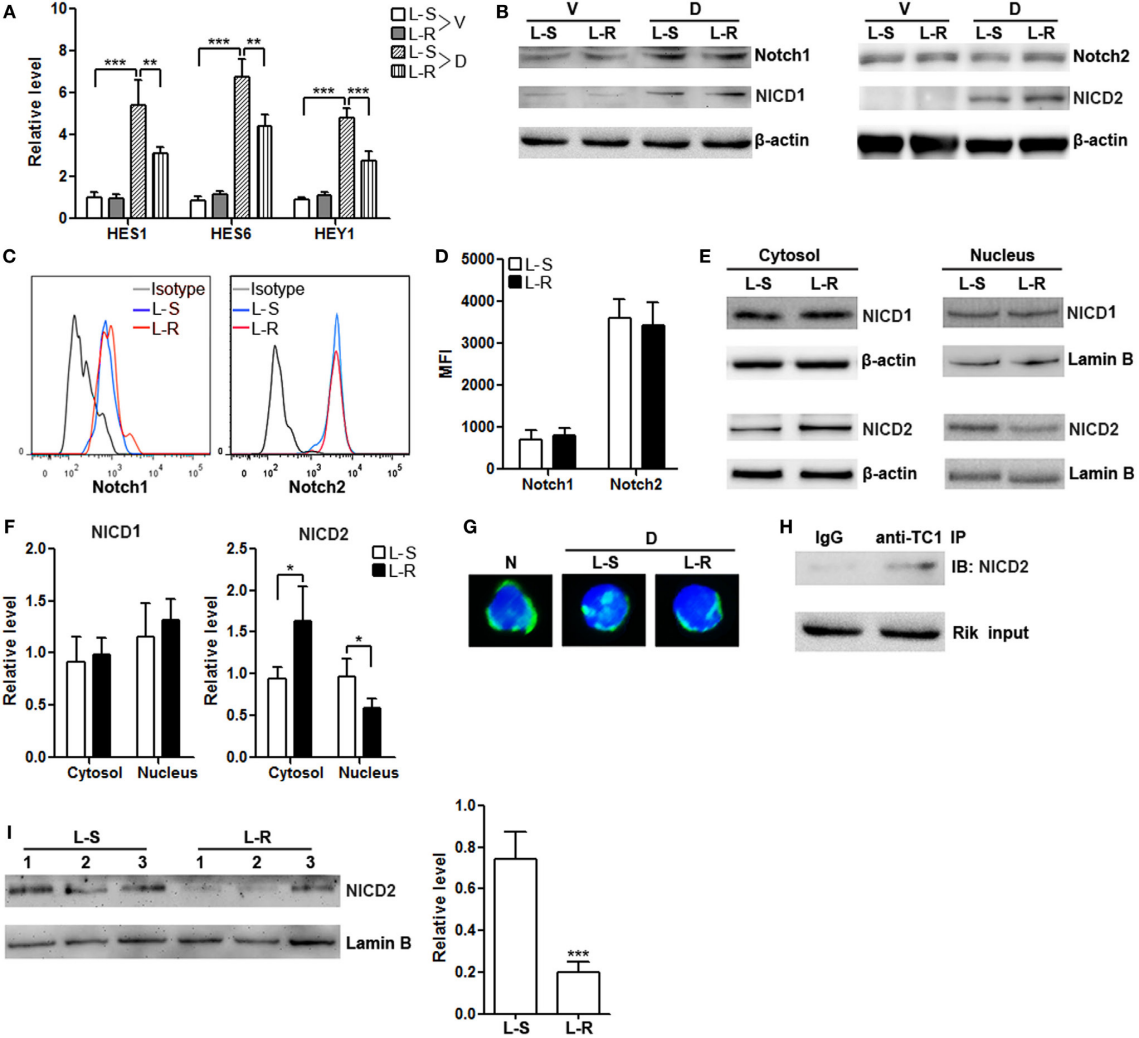
**Rik inhibits nuclear translocation of Notch2 intracellular domain (NICD2)**. **(A)** mRNA levels of HES1, HES6, and HEY1 in CD8^+^ T cells. Lentivirus-transduced CD8^+^ T cells were stimulated *in vitro* with immobilized delta-like 1 (DLL1) for 4 h before quantitative polymerase chain reaction. L-S: CD8^+^ T cells transduced with scramble lentiviruses. L-R: CD8^+^ T cells transduced with Rik-expressing lentiviruses. V: vehicle. D: DLL1. *N* = 3 per group. **(B)** Expression of Notch1, Notch2, NICD1, and NICD2 in the whole cell lysates after 1-h stimulation with DLL1. This is a representative of two independent experiments. **(C,D)** Cell surface expression of Notch1 and Notch2 after 4-h stimulation with DLL1. Representative histograms are shown in panel **(C)**, and statistics of mean fluorescent intensity is shown in panel **(D)**. *N* = 3 per group. **(E,F)** Expression of NICD1 and NICD2 in the cytosol and nucleus of CD8^+^ T cells after 1-h stimulation with DLL1. Representative Immunoblot images are shown in panel **(E)**, and statistical analysis is shown in panel **(F)**. *N* = 4 per group. **(G)** Fluorescent microscopy of Notch2. The polyclonal anti-Notch2 antibody recognized both intact Notch2 and NICD2. N: naïve CD8^+^ T cells. D: DLL1-stimulated CD8^+^ T cells. **(H)** Immunoprecipitation of Rik and detection of NICD2. This is a representative of two independent experiments. **(I)** Nuclear NICD2 levels in isolated intratumoral CD8^+^ T cells from 3 individual mouse of each group. Left panel: representative Immunoblot image. Right panel: statistics of nuclear NICD2 levels. L-S: mice receiving control CD8^+^ T cells. L-R: mice receiving Rik-overexpressing CD8^+^ T cells (**p < 0.01; ***p < 0.001).

The differential *in vitro* and *in vivo* effects of Rik on CD8^+^ T cells suggest that Rik might work on certain signal pathways other than TCR/CD3/CD28 signaling. Notch signaling has been considered crucial for cytotoxic T cell differentiation (23). Moreover, a previous paper implies that human TC1 blocks the entry of activated Notch2 into the nuclei of cancer stem cells (19). To test if this mechanism also underlies the *in vivo* effect of Rik on CD8^+^ T cell function, we firstly analyzed the expression of several North target genes such as HES1, HES6, and HEY1 in CD8^+^ T cells after stimulation with immobilized DLL1, which is a ligand for both Notch1 and Notch2. In the absence of DLL1, Rik-overexpressing CD8^+^ T cells and control CD8^+^ T cells expressed comparably low levels of HES1, HES6, and HEY1 (Figure 6A). In the presence of DLL1, Rik-overexpressing CD8^+^ T cells expressed lower HES1, HES6, and HEY1, in comparison with control CD8^+^ T cells (Figure 6A). However, we found that Rik overexpression did not significantly alter the expression of Notch and NICD in the whole cell lysates (Figures 6B–D). Rather, analysis of cytosolic and nuclear NICD revealed more cytosolic NICD2 and less nuclear NICD2 in Rik-overexpressing CD8^+^ T cells, while NICD1 distribution was not changed (Figures 6E,F). Immunofluorescent staining showed less staining of nuclear NICD2 in Rik-overexpressing CD8^+^ T cells (Figure 6G). Moreover, it seems that Rik might directly bind to NICD2 (Figure 6H), suggesting that the mechanism by which human TC1 modulates cancer stem cells might also be involved in Rik-mediated regulation of CD8^+^ T cell function. Notably, nuclear NICD2 level was also decreased in Rik-overexpressing CD8^+^ T cells *in vivo*, in comparison with control CD8^+^ T cells (Figure 6I).

### Antigen-Presenting Cells and TGF-β Induce Rik Expression in CD8^+^ T Cells

**Figure 7:**
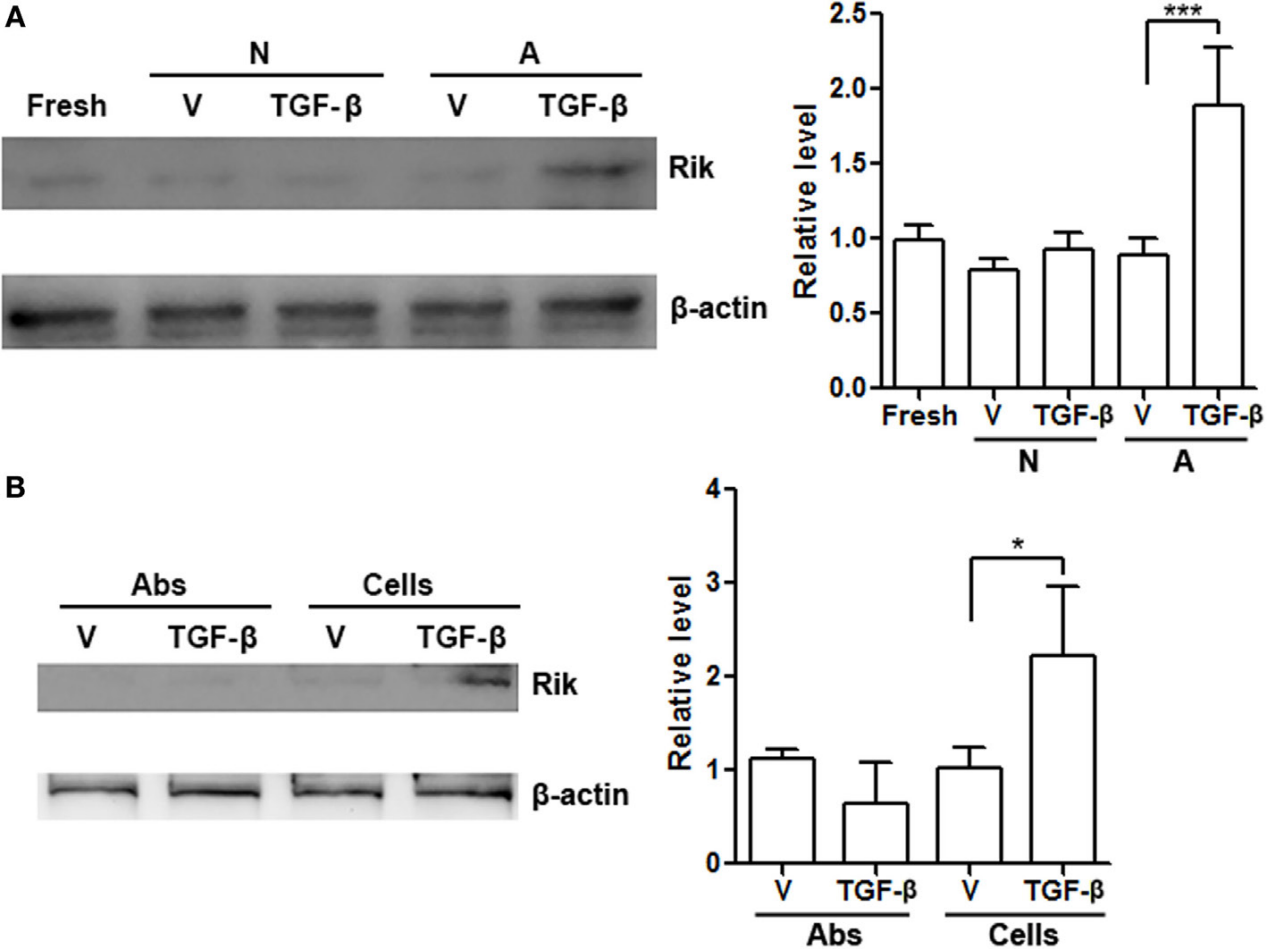
**Antigen-presenting cells and TGF-β are necessary for upregulation of Rik expression**. **(A)** Rik expression in CD8^+^ T cells after treatment. Left panel: representative Immunoblot image. Right panel: statistics. Fresh: freshly isolated CD8^+^ T cells. N: no activation. A: activation with BALB/c splenocytes depleted of T cells. V: phosphate-buffered saline (PBS). *N* = 3 per group. **(B)** Rik expression in CD8^+^ T cells after treatment with agonistic antibodies or BALB/c splenocytes. Left panel: representative Immunoblot image. Right panel: statistics. Abs: agonistic antibodies. Cells: BALB/c splenocytes. V: PBS. *N* = 3 per group (*p < 0.05; ***p < 0.001).

To roughly determine the possible extracellular factors that contributing to Rik expression, CD8^+^ T cells were cultured *in vitro* with BALB/c splenocytes depleted of T cells in the presence or absence of TGF-β. Splenocytes or TGF-β alone had no effect on Rik expression, while splenocytes plus TGF-β moderately enhanced Rik expression (Figure 7A). We then tested whether treatment with agonistic antibodies and TGF-β had similar effect on Rik induction. To our surprise, agonistic antibodies with TGF-β failed to upregulate Rik expression (Figure 7B), suggesting that CD3/CD28 signaling might not be sufficient for Rik expression. Taken together, our results implied that TGF-β worked together with unidentified antigen-presenting cell-derived factors to induce Rik expression in CD8^+^ T cells.

## Discussion

In this study, we identified the presence of Rik in intratumoral CD8^+^ T cells. Rik is the homolog of human TC1 which regulates various tumor cell proliferation and differentiation. Recently, it was reported to be involved in hematopoietic stem/progenitor cell expansion and hematopoiesis (21). Other tumor-or-stem cell-related proteins, such as members of Wnt (24, 25), Notch (23, 26), and Hedgehog (27, 28) families, as well as Myc (29, 30) and KLF4 (31, 32), have been previously shown to be essential for mature T cell activation and function. It is likely that T cell activation requires several tumor-or-stem cell-related signal pathways to modulate expansion and differentiation toward effector T cells. The upregulation of Rik in intratumoral CD8^+^ T cells therefore might be a consequence of the overall change of cell signaling in activated CD8^+^ T cells. Indeed, we found that Rik was predominantly expressed in the CD44^+^CD62L^−^ subpopulation of intratumoral CD8^+^ T cells. This subpopulation represents the activated/effector cells which possess potent antitumor activity. Interestingly, Rik expression was not upregulated in the splenic CD8^+^CD44^+^CD62L^−^ T cells, suggesting that Rik was induced by the tumor microenvironment, or Rik was expressed only in tumor-specific CD8^+^ T cells.

To our surprise, Rik overexpression did not impact agnostic antibody-induced CD8^+^ T cell activation. This result strongly suggests no involvement of Rik in CD3/CD28-mediated cell signaling. Another possibility is that CD3/CD28-mediated cell signaling was too strong to be overcome by the effect of Rik in the *in vitro* study, since the agonistic antibodies induced very robust activation. Our ongoing research is testing whether suboptimal activation could be influenced by Rik overexpression. However, *in vivo* experiments indicated the negative role of Rik on CD8^+^ T cell function. The differential results of *in vitro* and *in vivo* study imply that the effect of Rik depends on several tissue-or-tumor-associated factors. It would be possible that *in vitro* settings lacked these necessary factors so the effect of Rik could not be seen. Indeed, in the following study, we identified the role of Notch signaling in Rik-mediated T cell regulation. So, it is likely that *in vitro* stimulated CD8^+^ T cells do not have sufficient Notch signaling for Rik to inhibit.

Interestingly, a recent study demonstrates the suppressive effect of human TC1 on Notch2 signaling in liver cancer stem cells (19). Moreover, the role of Notch2 in regulating CD8^+^ T cell function has been extensively studied. It is reported that antigen-presenting cells, especially dendritic cells, express Notch ligands Jagged1 and Delta-like 4, whereas naive CD8^+^ T cells express Notch2 (23). Inhibition of Notch during activation reduced frequencies of IFN-γ-, TNF-α-, and granzyme B-producing CD8^+^ T cells (23). More importantly, it has been shown that signaling by Notch2 but not Notch1 in CD8^+^ T cells is required for antitumor cytotoxic T lymphocyte responses, because specific knockout of Notch2 in CD8^+^ T cells decreases antitumor cytotoxic T lymphocyte responses while specific knockout of Notch1 does not (33). Notably, in HCC sites, the sources of Notch ligands might include antigen-presenting cells (20), HCC cells (34), or vascular endothelial cells (35, 36). Intratumural CD8^+^ T cells might engage Notch ligands through their surface Notch1 and Notch2. These reports led us to explore the potential mechanism of Rik’s effect. Similar to what was observed in liver cancer stem cells, reduced nuclear translocation of NICD2 was found in Rik-overexpressing CD8^+^ T cells upon *in vitro* stimulation with Notch2 ligand DLL1. And decreased nuclear NICD2 was also seen in Rik-overexpressing CD8^+^ T cells *in vivo*. Taken together, Rik might modulate CD8^+^ T cell function through the same mechanism as it works on liver cancer stem cells.

Notably, we found that Rik expression could be induced by concurrent presence of T cell-free splenocytes and TGF-β. Although this experiment was actually a one-way mixed lymphocyte reaction rather than a tumor antigen-specific immune response, we think it still provides important information on the expression of Rik in CD8^+^ T cells. It should be noted that agonistic antibodies with TGF-β was unable to induce Rik expression, suggesting that splenocytes might provide extra signals in addition to TCR/CD28 signaling to trigger Rik expression. The exact identity of these signals, and whether they are cell surface proteins or soluble mediators, is still unknown. To more accurately characterize the role of antigen-presenting cells in the induction of Rik, genetically engineered mouse strains such as OT-I mouse, and special tumor cell lines such as OVA (257-264)-expressing mouse HCC cell lines, will be very helpful. However, we are currently unable to acquire these materials. Our lab is establishing several mouse HCC cell lines expression OVA, and we hope to buy the OT-I mouse strain in future.

With regard to TGF-β, a previous research also showed that TGF-β upregulates human TC1 expression in colon cancer (14). TGF-β has long been considered a crucial contributing factor to carcinogenesis of HCC (37) and a suppressive agent for CD8^+^ T cells (38). Besides, upregulated TGF-β expression is observed in HCC (39–41). Most hepatocarcinoma cells are able to synthesize and secrete TGF-β continually by themselves. Therefore, it is very likely that in the HCC sites, HCC-derived TGF-β collaborates with antigen-presenting cells to enhance Rik expression in tumor-specific CD8^+^ T cells. To test this hypothesis, our lab is trying to isolate antigen-presenting cells, particularly dendritic cells from tumor implants so as to coculture them with intratumoral CD8^+^ T cells in the presence of TGF-β. However, it is very difficult to do so due to the limited abundance of intratumoral dendritic cells. Chemical-induced HCC model might be an alternative. Another possibility is that tumor cells, which present their MHC-I–antigen complex and express TGF-β, could effectively induce Rik expression in tumor-specific CD8^+^ T cells. Our lab will also test this hypothesis in future.

Besides Notch2 signaling, human TC1 has been indicated to regulate Wnt/β-catenin (15, 42) and NF-κB pathways (43). These pathways are also important for CD8^+^ T cell activity. The potential role of Rik in modulating these pathways in CD8^+^ T cells should be elucidated in further research. In conclusion, our study unveils a novel mechanism by which anti-HCC activity of CD8^+^ T cells is impaired. Our data might shed light on development of new therapeutic approaches to promote anti-HCC immunity.

## Author Contributions

KD, L-hY, and Y-aJ conceived the study, analyzed the data, and wrote the manuscript. KD, LH, Y-bH, and Z-bC performed the research. All the authors read and approved the final manuscript.

## Conflict of Interest Statement

The authors declare that the research was conducted in the absence of any commercial or financial relationships that could be construed as a potential conflict of interest. The reviewer XR and handling editor declared their shared affiliation, and the handling editor states that the process nevertheless met the standards of a fair and objective review.
