# Supplementary material for: 1810011o10 Rik Inhibits the Antitumor Effect of Intratumoral CD8+ T Cells through Suppression of Notch2 Pathway in a Murine Hepatocellular Carcinoma Model
Source: Front Immunol. 2017 Mar 22;8:320. doi: 10.3389/fimmu.2017.00320 (PMC5360711; doi:10.3389/fimmu.2017.00320)
Supplement: Supplementary file 1 [file Data_Sheet_1.DOCX]

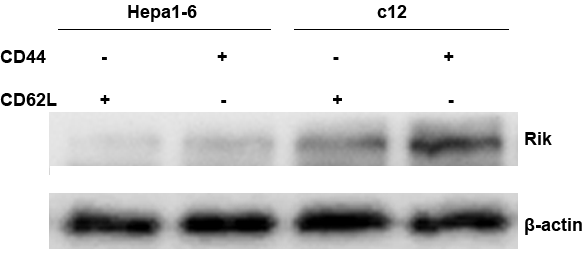


**Supplementary figure S1. Expression of Rik in CD8^+^CD44^-^CD62L^+^ and CD8^+^CD44^+^CD62L^-^ T cells isolated from Hepa1-6 or c12 tumor implants.**

**
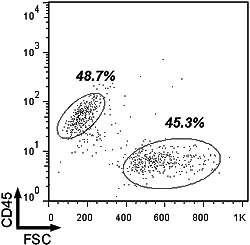
**

**Supplementary figure S2. Staining of CD45 distinguishes tumor cells after co-culture with T cells.** CD45^-^ tumor cells were subject to apoptosis assay using Annexin V staining.


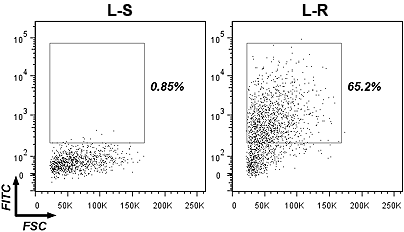


**Supplementary figure S3. Expression of Rik after lentiviral transduction.** Cells were stained with 10 µg/ml anti-Rik antibody before incubation with 5 µg/ml FITC-conjugated goat anti-rabbit IgG, according to intracellular staining protocol described in “Materials and Methods”. L-S: transduction with lentiviruses containing scramble sequence. L-R: transduction with lentiviruses containing Rik sequence.
